# Combined LXR and RXR Agonist Therapy Increases ABCA1 Protein Expression and Enhances ApoAI-Mediated Cholesterol Efflux in Cultured Endothelial Cells

**DOI:** 10.3390/metabo11090640

**Published:** 2021-09-18

**Authors:** Kun Huang, Hanjoong Jo, Jing Echesabal-Chen, Alexis Stamatikos

**Affiliations:** 1 Department of Food, Nutrition, and Packaging Sciences, Clemson University, Clemson, SC 29634, USA; kunh@g.clemson.edu (K.H.); jchen11@clemson.edu (J.E.-C.); 2 Coulter Department of Biomedical Engineering, Georgia Institute of Technology and Emory University, Atlanta, GA 30322, USA; hjo@emory.edu

**Keywords:** atherogenesis, cardiovascular disease, endothelial activation, endothelial dysfunction, oxysterols, reverse cholesterol transport, vascular inflammation

## Abstract

Endothelial ABCA1 expression protects against atherosclerosis and this atheroprotective effect is partially attributed to enhancing apoAI-mediated cholesterol efflux. ABCA1 is a target gene for LXR and RXR; therefore, treating endothelial cells with LXR and/or RXR agonists may increase ABCA1 expression. We tested whether treating cultured immortalized mouse aortic endothelial cells (iMAEC) with the endogenous LXR agonist 22(R)-hydroxycholesterol, synthetic LXR agonist GW3965, endogenous RXR agonist 9-cis-retinoic acid, or synthetic RXR agonist SR11237 increases ABCA1 protein expression. We observed a significant increase in ABCA1 protein expression in iMAEC treated with either GW3965 or SR11237 alone, but no significant increase in ABCA1 protein was observed in iMAEC treated with either 22(R)-hydroxycholesterol or 9-cis-retionic acid alone. However, we observed significant increases in both ABCA1 protein expression and apoAI-mediated cholesterol efflux when iMAEC were treated with a combination of either 22(R)-hydroxycholesterol and 9-cis-retinoic acid or GW3965 and SR11237. Furthermore, treating iMAEC with either 22(R)-hydroxycholesterol and 9-cis-retinoic acid or GW3965 and SR11237 did not trigger an inflammatory response, based on VCAM-1, ICAM-1, CCL2, and IL-6 mRNA expression. Based on our findings, delivering LXR and RXR agonists precisely to endothelial cells may be a promising atheroprotective approach.

## 1. Introduction

Atherosclerosis leads to more deaths than any other disease both in the United States and globally because it is the leading cause of myocardial infarctions and ischemic strokes [1,2]. Atherosclerosis is mainly caused by cholesterol accumulating in arteries [3]. Unfortunately, the most common treatment for atherosclerosis, oral statin drugs, work to decrease cholesterol levels systemically instead of reducing cholesterol specifically from arteries, and statins are only partially effective against atherosclerosis and preventing myocardial infarctions and ischemic strokes [4,5,6,7]. Therefore, a better therapy for atherosclerosis and for the prevention of future myocardial infarctions and ischemic strokes may be treatments that are able to safely and effective remove cholesterol precisely from arteries.

Historically, the consequence of intimal arterial macrophages becoming cholesterol-filled has traditionally been considered the prime reason for atherogenesis and atherosclerosis progression [8,9,10]. However, recent evidence now strongly supports arterial endothelial cell cholesterol accumulation to both trigger and exacerbate atherosclerosis [11,12]. If arterial endothelial cells becoming cholesterol-filled does drive atherosclerosis, then therapies geared to deplete cholesterol from these cholesterol-laden endothelial cells may successfully treat atherosclerosis. A potential treatment for achieving this may be liver X receptor (LXR) and retinoid X receptor (RXR) agonist therapy. The reason for this is because the nuclear receptors LXR and RXR can form heterodimers when activated and these heterodimers are able to activate ATP binding cassette subfamily A member 1 (ABCA1) expression [13,14]. LXR functions to regulate lipid metabolism, while RXR aids in mediating cellular metabolism and cell differentiation [15,16]. ABCA1 is a transporter that functions to remove excess cholesterol from cells when it interacts with the cholesterol acceptor protein known as apolipoprotein A-I (apoAI) [17,18,19,20]. Hence, identifying which combination of LXR and RXR agonists induce the largest increases in ABCA1 protein expression and apoAI-mediated cholesterol efflux in cultured endothelial cells should be examined.

While LXR (and RXR) agonists have already been shown to increase ABCA1 expression in cultured endothelial cells [21,22,23], LXR agonists have also been shown to alter inflammation in cultured endothelial cells, too. For instance, certain synthetic LXR agonists appear to suppress inflammation in endothelial cells. However, endogenous LXR agonists appear to induce inflammation in endothelial cells [23,24]. Moreover, while data are lacking to determine whether RXR agonists alter inflammation specifically in endothelial cells, data do show that RXR agonists suppress inflammation in other cultured cells [25,26,27]. The concept of endothelial cell inflammation is extremely important to atherosclerosis therapies, as inflamed endothelium is known to be a driver for atherosclerosis, too [28,29]. Therefore, if LXR and/or RXR agonists are to be used as a form of atherosclerosis therapy, they cannot induce endothelial cell inflammation, as this may result in a pro-atherogenic effect instead of an anti-atherogenic effect. Thus, examining the potential inflammatory impact LXR/RXR agonist treatment may have on endothelial cells should be directly tested.

The purpose of this work is to test whether exposing endothelial cells to LXR and/or RXR agonists increases ABCA1-dependent cholesterol efflux in these cells without stimulating inflammation. In this study, we examined whether treating cultured immortalized mouse aortic endothelial cells (iMAEC) [30] with endogenous and synthetic LXR and RXR agonists increases ABCA1 protein expression. We observed that treating iMAEC with the endogenous LXR agonist 22(R)-hydroxycholesterol or endogenous RXR agonist 9-cis-retinoic acid alone failed to significantly increase ABCA1 protein, while treatment with the synthetic LXR agonist GW3965 or synthetic RXR agonist SR11237 alone was successful in significantly increasing ABCA1 protein expression in iMAEC. However, combined therapy of 22(R)-hydroxycholesterol and 9-cis-retinoic acid increased both ABCA1 protein expression and apoAI-mediated cholesterol efflux in iMAEC, as did the combined treatment of GW3965 and SR11237. Furthermore, treatment of either 22(R)-hydroxycholesterol/9-cis-retinoic acid or GW3965/SR11237 did not result in significantly increasing pro-inflammatory gene expression in iMAEC. Based on our results, we conclude that endothelial-specific LXR/RXR agonist therapy may be implemented as a potential atherosclerosis treatment.

## 2. Results

### 2.1. iMAEC Express ABCA1, LXR, and RXR Proteins, and Are Capable of Effluxing Cholesterol to ApoAI

**Figure 1:**
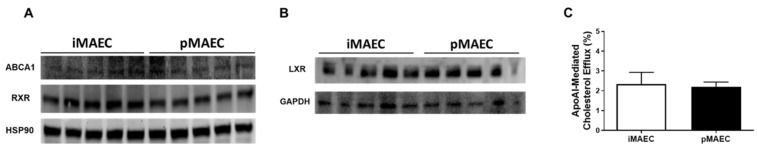
Detection of ABCA1, RXR, and LXR protein expression and quantifying cholesterol effluxed to apoAI in immortalized mouse aortic endothelial cells (iMAEC). (**A**,**B**) Protein detected via immunoblotting in iMAEC and primary mouse aortic endothelial cells (pMAEC; positive control) cultured in basal conditions for either ABCA1 and RXR (**A**) or LXR (**B**). Five replicates per cell type. HSP90 (**A**) and GAPDH (**B**) are housekeeping proteins. (**C**) ApoAI-mediated cholesterol efflux measured in serum-starved, [^3^H]cholesterol-loaded iMAEC and pMAEC. Two independent experiments with three biological replicates per treatment for each experiment. Data are mean ± SEM.

To determine whether cultured iMAEC [30] could be used as a suitable cell line for our proposed experiments, we first needed to test whether iMAEC express ABCA1, RXR, and LXR protein. We used immunoblotting to analyze protein expression in iMAEC and used cultured primary mouse aortic endothelial cells (pMAEC) as a positive control, as primary endothelial cells are known to express ABCA1, LXR, and RXR [31,32,33]. Our results show detection of ABCA1, RXR, and LXR protein in iMAEC cultured in basal conditions (Figure 1A,B). To assess functionality of ABCA1 protein in iMAEC, we also performed cholesterol efflux assays using apoAI as the cholesterol acceptor, and used pMAEC as our positive control [34,35,36,37]. Our results demonstrate that iMAEC are capable of ABCA1-dependent cholesterol efflux (Figure 1C), since apoAI exclusively participates in cholesterol efflux through interaction with ABCA1 [38]. Therefore, we conclude that iMAEC is an appropriate cell line to use for our apoAI-mediated cholesterol efflux assays involving treatment with LXR and RXR agonists.

### 2.2. Synthetic LXR and RXR Agonists, but Not Endogenous LXR and RXR Agonists, Increase ABCA1 Protein Expression in iMAEC

**Figure 2:**
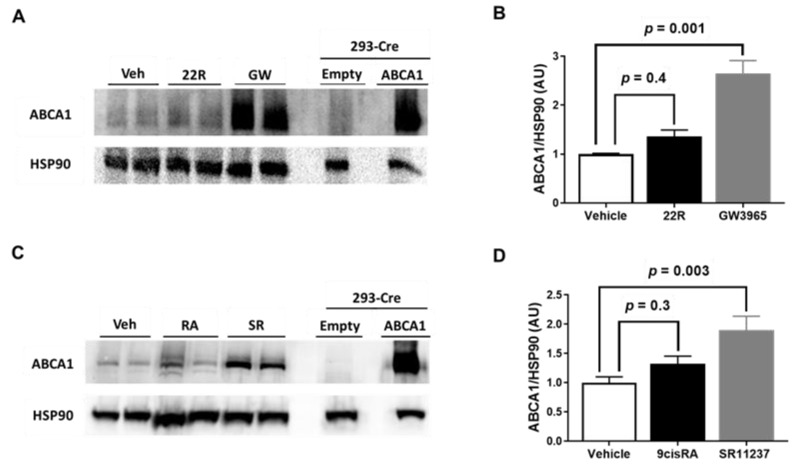
Effect of LXR and RXR agonist treatment on ABCA1 protein expression in immortalized mouse aortic endothelial cells (iMAEC). (**A**) iMAEC were cultured in basal conditions and treated with either vehicle only (Veh), 22(R)-hydroxycholesterol (22R), or GW3965 (GW), and ABCA1 protein from iMAEC lysates were assessed by immunoblotting. (**B**) Quantification of ABCA1 protein in representative immunoblot (**A**) and two other immunoblots (three independent treatments; each with two biological replicates per respective treatment). (**C**) iMAEC cultured in basal conditions and treated with Veh, 9-cis-retinoic acid (RA), or SR11237 (SR), and ABCA1 protein from iMAEC lysates were analyzed via immunoblotting. (**D**) Quantification of ABCA1 protein in representative immunoblot (**C**) and two other immunoblots (three independent treatments; each with two biological replicates per respective treatment). (**A**,**C**) 293-Cre cells transfected with empty vector plasmid (technical negative control) or ABCA1-expressing plasmid (technical positive control). HSP90 is the housekeeping protein. (**B**,**D**) AU, arbitrary units; data are mean ± SEM.

LXR and RXR agonists are attractive candidates for atherosclerosis therapy, since they are capable of inducing ABCA1 expression [13,14]. Therefore, we wanted to directly test whether exposing iMAEC to either LXR or RXR agonists alone upregulated ABCA1 protein expression. We selected the oxysterol 22(R)-hydroxycholesterol and RXR agonist 9-cis-retinoic acid as our endogenous LXR and RXR agonists, respectively, as previous reports have shown treating cultured endothelial cells with these agonists increases ABCA1 mRNA expression [22]. For our synthetic LXR agonist, we chose GW3965, as prior findings have observed GW3965 treatment is able to upregulate ABCA1 and enhance cellular cholesterol efflux [39,40]. Moreover, GW3965 appears to be a specific LXR agonist when compared to another potent synthetic LXR agonist, T0901317, which has also been recognized to act as a pregnane X receptor agonist [41]. We chose SR11237 as our synthetic RXR agonist, since this synthetic RXR agonist does appear to be a truly specific RXR agonist [15,42,43]. For our results, we show that incubating iMAEC with either 22(R)-hydroxycholesterol or 9-cis-retinoic acid alone failed to increase ABCA1 protein expression, while exposing iMAEC to either GW3965 or SR11237 alone resulted in significantly increasing ABCA1 protein expression (Figure 2A–D). These results imply that exposing endothelial cells to synthetic LXR or RXR agonists alone results in robust upregulation of ABCA1 protein that fails to occur when endothelial cells are exposed to either endogenous LXR agonists or endogenous RXR agonists alone.

### 2.3. Combining LXR/RXR Agonist Treatments Increases Both ABCA1 Protein and ApoAI-Mediated Cholesterol Efflux in iMAEC

**Figure 3:**
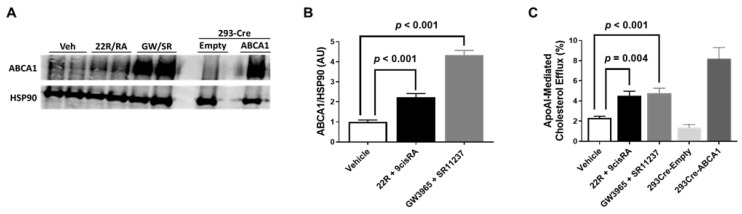
Effect of combining LXR and RXR agonist treatments on ABCA1 protein expression and apoAI-mediated cholesterol efflux in immortalized mouse aortic endothelial cells (iMAEC). (**A**) iMAEC were cultured in basal conditions and treated with either vehicle only (Veh), 22(R)-hydroxycholesterol and 9-cis-retinoic acid (22R/RA), or GW3965 and SR11237 (GW/SR). 293-Cre cells were transfected with either empty vector plasmid (technical negative control) or ABCA1-expressing plasmid (technical positive control). ABCA1 protein from cell lysates were assessed via immunoblotting. HSP90 is the housekeeping protein. (**B**) Quantification of ABCA1 in representative immunoblot (**A**) and two other immunoblots for iMAEC (three independent treatments; each with two biological replicates per respective treatment). AU, arbitrary units; data are mean ± SEM. (**C**) ApoAI-mediated cholesterol efflux measured in serum-starved, [^3^H]cholesterol-loaded iMAEC treated with vehicle only, 22R and 9-cis-retinoic acid, or GW3965 and SR11237. ApoAI-mediated cholesterol efflux was also measured in serum-starved, [^3^H]cholesterol-loaded 293-Cre cells transfected with empty vector plasmid (technical negative control) or ABCA1-expressing plasmid (technical positive control). Three independent experiments with three biological replicates per treatment for each experiment. Data are mean ± SEM.

Previous literature has shown ABCA1 mRNA expression to be increased even further in cultured endothelial cells when exposed to 22(R)-hydroxycholesterol and 9-cis-retinoic acid simultaneously when compared to incubation of either of these agonists alone [22]. Furthermore, combined treatment of 22(R)-hydroxycholesterol and 9-cis-retinoic acid has also shown to robustly increase ABCA1 protein in cultured endothelial cells, too [21]. Therefore, we incubated iMAEC with either the endogenous LXR/RXR agonists 22(R)-hydroxycholesterol/9-cis-retinoic acid simultaneously or the synthetic LXR/RXR agonists GW3965/SR11237 simultaneously. In these experiments, we observed a significant increase in ABCA1 protein expression in both groups of iMAEC exposed to either the endogenous or synthetic LXR/RXR agonists when compared to vehicle-treated control iMAEC (Figure 3A,B). When we measured apoAI-mediated cholesterol efflux in these treated cells, we also observed significant increases in apoAI-mediated cholesterol efflux in the iMAEC incubated with the LXR/RXR agonists when compared to the vehicle-treated control iMAEC group (Figure 3C). Our results suggest that there is an additive (or synergistic) effect that occurs when endothelial cells are exposed to LXR and RXR agonists simultaneously, which results in enhancing ABCA1-dependent cholesterol efflux within endothelial cells.

### 2.4. Combined LXR/RXR Agonist Therapy in iMAEC Fails to Alter Pro-Inflammatory Gene Expression

**Figure 4:**
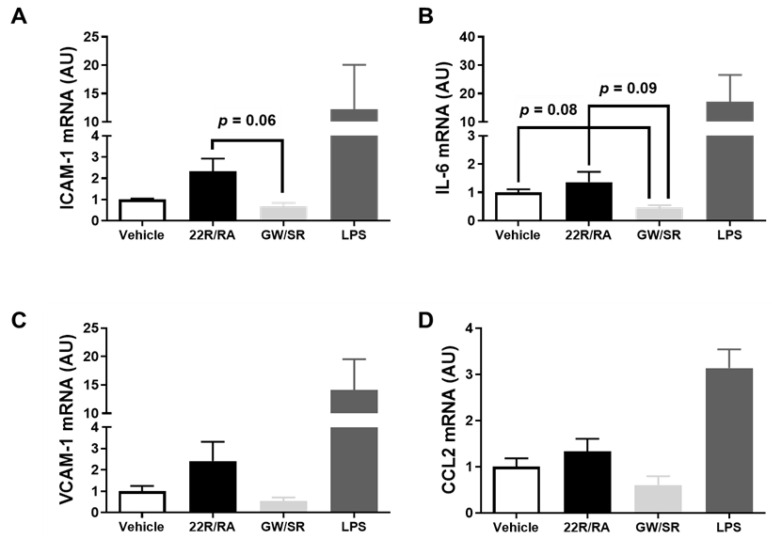
Effect of combining LXR and RXR agonist treatments on pro-inflammatory gene expression in immortalized mouse aortic endothelial cells (iMAEC). (**A**–**D**) iMAEC were cultured in basal conditions and either vehicle treated, treated with 22(R)-hydroxycholesterol and 9-cis-retinoic acid (22R/RA), or treated with GW3965 and SR11237 (GW/SR), and RT-qPCR was used to measure ICAM-1 (**A**), IL-6 (**B**), VCAM-1 (**C**), and CCL2 (**D**) mRNA expression in the treated iMAEC. LPS-treated iMAEC acted as a positive control. Three independent experiments with two biological replicates per treatment for each experiment. AU, arbitrary units; data are mean ± SEM.

There are data which suggest 22(R)-hydroxycholesterol and other oxysterols are pro-inflammatory when they are introduced to endothelial cells [23,24]. However, while these types of endogenous LXR agonists are thought to be pro-inflammatory to endothelium, interestingly, synthetic LXR agonists, such as GW3965, have been shown to demonstrate anti-inflammatory properties in cultured endothelial cells [23,24]. Moreover, 9-cis-retinoic acid has exhibited anti-inflammatory effects in various cultured cells [25,26,27]. Due to these intriguing findings, we wanted to measure pro-inflammatory gene expression in iMAEC exposed to LXR/RXR agonists, since inflammation is a well-established driver for atherosclerosis [3,28,29,44]. When comparisons were made among vehicle-treated control iMAEC, iMAEC treated with 22(R)-hydroxycholesterol/9-cis-retinoic acid, and iMAEC treated with GW3965/SR11237, there were no significant differences in mRNA expression of the pro-inflammatory adhesion molecules and cytokines ICAM-1, VCAM-1, IL-6, and CCL2 (i.e., MCP-1) [45] (Figure 4A–D), which is imperative, as all of these measured genes are recognized as playing a prominent role in atherosclerosis development and vascular inflammation [46]. Based on these results, treating endothelial cells with LXR and RXR agonists simultaneously does not appear to influence vascular inflammation.

## 3. Discussion

In this study, we wanted to directly test whether treating cultured endothelial cells with LXR and RXR agonists upregulates ABCA1 protein expression. However, we first needed to identify a correct cell type to robustly test for this. While pMAEC would have been a suitable cell type to utilize for our experiments, pMAEC are notorious for being finicky, become senescent after a low passage number, are tedious and difficult to isolate from mice, and need to be characterized after culture to assess fibroblast and vascular smooth muscle cell contamination, which would negatively interfere with study findings [47,48]. Moreover, while there are commercial vendors now that sell pMAEC, they are expensive to purchase. Ideally, an immortalized aortic endothelial cell line may remedy these problems, but there is no such cell line that has been shown in published literature to express ABCA1, LXR, and RXR protein, or has been shown to demonstrate apoAI (i.e., ABCA1-dependent) mediated cholesterol efflux. However, in our study, we characterized iMAEC to definitively show that this cell line does express ABCA1, LXR, and RXR protein. Furthermore, we demonstrated that these three proteins are functional via performing cholesterol efflux assays using apoAI as the cholesterol acceptor and demonstrating that treating these cells with LXR and RXR agonists does upregulate protein expression of its target gene ABCA1 [49]. When treating iMAEC with LXR and RXR agonists, both the synthetic LXR agonist GW3965 and the synthetic RXR agonist SR11237 were able to significantly increase ABCA1 protein expression alone, but the endogenous LXR agonist 22(R)-hydroxycholesterol and endogenous RXR agonist 9-cis-retinoic acid alone failed to significantly increase ABCA1 protein in iMAEC. However, when iMAEC were treated with 22(R)-hydroxycholesterol and 9-cis-retinoic acid simultaneously, this resulted in significantly increasing ABCA1 protein expression. Furthermore, as expected, treating iMAEC with GW3965 and SR11237 simultaneously also resulted in significantly increasing ABCA1 protein. Both types of LXR/RXR agonist treatments were shown to significantly increase apoAI-mediated cholesterol efflux in iMAEC, too.

While lipid accumulation drives atherosclerosis, vascular inflammation is known as a hallmark for atherosclerosis as well [28,29]. Moreover, the oxysterol and endogenous LXR agonist 22(R)-hydroxycholesterol has previously been shown to be pro-inflammatory in cultured endothelial cells [24]. Therefore, we measured pro-inflammatory mRNA expression in iMAEC treated with LXR/RXR agonists. Interestingly, there was no significant difference in the mRNA expression levels of ICAM-1, VCAM-1, CCL2, or IL-6 among vehicle-treated iMAEC, iMAEC treated with 22(R)-hydroyxcholesterol/9-cis-retinoic acid, or iMAEC treated with GW3965/SR11237. Since 9-cis-retinoic acid has exhibited anti-inflammatory effects in various cultured cells [25,26,27], it is possible that simultaneous treatment of 22(R)-hydroxycholesterol with 9-cis-retinoic acid may have potentially counteracted the pro-inflammatory response that has been shown to occur in endothelial cells from oxysterol exposure [24]. However, another possibility is iMAEC losing some primary cell physiological properties that may have potentially accounted for the lack of a pro-inflammatory response observed when iMAEC were exposed to the endogenous LXR agonist 22(R)-hydroxycholesterol, which is in contrast to primary cultured endothelial cells exposed to this oxysterol [24]. We also want to acknowledge that if iMAEC have lost certain primary cell physiological properties, then this may potentially account for why ABCA1 expression was not significantly increased in this cell line from either 22(R)-hydroxycholesterol or 9-cis-retinoic acid treatment alone, as this result differs from previous reports that observed significant increases in ABCA1 expression when primary cultured endothelial cells were treated with these agonists alone [22]. Therefore, future studies involving treatment with 22(R)-hydroxycholesterol and/or 9-cis-retinoic acid in iMAEC in parallel with pMAEC should be conducted to distinguish any noticeable differences in ABCA1-dependent cholesterol efflux and inflammation between these two cell types from these treatments.

A prerequisite for atherosclerosis is cholesterol accumulation in arteries [50]. While it has historically been thought that cholesterol accumulation in macrophages drives atherosclerosis [8,9,10], there is now strong support for endothelial cell cholesterol accumulation triggering atherosclerosis, too [11,12]. The transporter ABCA1 is thought to be primarily responsible for removing cholesterol from numerous cell types, including both macrophages and endothelial cells [17,18,19,20]. Data have shown that ablating endothelial ABCA1 expression exacerbates atherosclerosis in mice [46], while overexpressing ABCA1 in endothelial cells is atheroprotective in mice [34]. However, in the latter study, it is important to emphasize that germline transgenesis was used to generate the endothelial-specific ABCA1 overexpressing mice [34], and this type of approach is not applicable to any type of atheroprotective therapy geared for humans. Fortunately, other strategies to upregulate ABCA1 protein in endothelial cells, such as using LXR and RXR agonists, may prove to be effective treatments for atherosclerosis.

In our study, we used cultured endothelial cells and thus we acknowledge that a limitation of our findings is that systemic administration of LXR and RXR agonists will most likely result in poor uptake of LXR/RXR agonists within endothelial cells in vivo. Caution is also warranted for administering LXR agonists systemically, as they are prone to causing hepatic steatosis and hypertriglyceridemia via activating genes involved in de novo lipogenesis [51]. Since both of these conditions may (at least indirectly) influence or aggravate atherosclerosis [52,53,54], a therapeutic strategy should be implemented to allow endothelial cells to be precisely targeted. This proposed approach should allow for an increase in LXR/RXR agonist endothelial cell uptake in vivo while simultaneously prevent LXR/RXR agonists to be internalized by other cells, such as the liver. A potential strategy that may be implemented would involve filling nanoparticles with LXR/RXR agonists and have these nanoparticles designed to precisely target inflamed endothelial cells. Indeed, this approach has been previously successful when using small RNA-filled nanoparticles that were precisely designed to target inflamed endothelium [55,56]. Since highly-inflamed endothelial cells reside in regions that are either atheroprone or have established atherosclerosis [57], filling nanoparticles with LXR/RXR agonists which precisely target these inflamed endothelial cells may be able to both treat and prevent atherosclerosis. Future studies should focus on generating these types of nanoparticles and first testing potential toxicity and effectiveness in vitro, then move onto testing safety and efficacy in atherogenic animal models.

In conclusion, our results show that the synthetic RXR agonist SR11237 and the synthetic LXR agonist GW3965 alone significantly increase ABCA1 protein expression in iMAEC, while the endogenous RXR agonist 9-cis-retinoic acid and the oxysterol and endogenous LXR agonist 22(R)-hydroxycholesterol alone failed to significantly increase ABCA1 protein in cultured endothelial cells. However, the combination of 22(R)-hydroxycholesterol/9-cis-retinoic acid significantly increased both ABCA1 protein expression and apoAI-mediated cholesterol efflux in iMAEC, as did the combination of GW3965/SR11237. For our gene expression results, there was no significant difference among mRNA expression levels for the pro-inflammatory genes IL-6, CCL2, VCAM-1, and ICAM-1 in iMAEC treated with LXR/RXR agonists. Future studies should determine whether the combination of GW3965/SR11237 is superior to other LXR/RXR agonist combinations not tested, as it may be possible that the combination of 22(R)-hydroxycholesterol/SR11237 and/or GW3965/9-cis-retinoic acid may increase ABCA1-dependent cholesterol efflux more than GW3965/SR11237, so these combinations in addition to other LXR/RXR-agonist [15,58] combinations should be directly tested. Furthermore, a dose–response curve for LXR/RXR-agonist therapy should be implemented to determine the maximum amount of ABCA1 upregulation and increase in apoAI-mediated cholesterol efflux that can occur without triggering toxicity in cultured endothelial cells. After identifying the best LXR/RXR combination to augment ABCA1-dependent cholesterol efflux without eliciting toxicity, future directions should be devoted to identifying an efficient delivery system that precisely delivers LXR/RXR agonists into the endothelium of atheroprone and atherosclerotic lesions and test whether this delivery method is both safe and effective against treating and/or preventing atherosclerosis.

## 4. Materials and Methods

### 4.1. Cell Culture Maintenance

Immortalized mouse aortic endothelial cells (iMAEC) [30], primary mouse aortic endothelial cells (pMAEC), and 293-Cre cells [59] were cultured in standard growth medium consisting of high-glucose Dulbecco’s Modified Eagle’s Medium (DMEM; Corning, New York, NY, USA), FB essence (10%; VWR Life Science Seradigm, Radnor, PA, USA), and penicillin/streptomycin (1%; Corning). The medium for iMAEC and 293-Cre cells was also supplemented with the antibiotic G418 (500 μg/mL; VWR Life Science Seradigm) [30,31,35,59]. pMAEC were purchased from Cell Biologics Inc. (Chicago, IL, USA) and were used between passages two and six before being discarded, while the iMAEC were provided by Dr. Hanjoong Jo [30]. For cell maintenance, all cells were maintained in 10 cm tissue culture dishes and placed in an incubator set at 37 °C and 5% CO_2_, with standard growth medium being replenished every two to three days.

### 4.2. Treating Cultured Cells

For protein expression related to iMAEC characterization, we cultured iMAEC and pMAEC in basal conditions in six well tissue culture plates, allowed cells to reach 70–80% confluency, harvested protein from these cells, and used cell lysates for western blotting experiments to characterize iMAEC, with the pMAEC acting as a positive control. For LXR and RXR agonist treatments involving assessing iMAEC protein expression, we first plated iMAEC into six well tissue culture plates, and then allowed the cells to grow in standard growth medium. Once iMAEC reached 70–80% confluency, we rinsed the cells with PBS (Corning), cultured iMAEC in basal conditions, and treated these cells with either vehicle-only or the following LXR and RXR agonists (all at a 10 μM final concentration) for 24 h: endogenous LXR agonist and oxysterol 22(R)-hydroxycholesterol (Sigma-Aldrich, St. Louis, MO, USA); synthetic LXR agonist GW3965 (Sigma-Aldrich); endogenous RXR agonist 9-cis-retinoic acid (Sigma-Aldrich); synthetic RXR agonist SR11237 (Sigma-Aldrich). After treatments, medium containing either vehicle or the LXR/RXR agonists were removed, cells rinsed with PBS, and iMAEC refed with standard growth medium for another 24 h, before harvesting iMAEC and using the cell lysates for Western blots. A technical control for our experiments when measuring ABCA1 protein expression in LXR and RXR agonist treated iMAEC were transfected 293-Cre cells [59]. We used these cells because they are derived from the HEK293 cell line which normally does not express ABCA1 protein [60]. We cultured 293-Cre cells in six well dishes in basal conditions and transfected these cells with jetOPTIMUS transfection reagent (Polyplus, New York, NY, USA) by using either a non-expressing empty vector control plasmid (technical negative control) (VectorBuilder Inc., Chicago, IL, USA) or a plasmid able to constitutively express ABCA1 protein (technical positive control) (VectorBuilder Inc.). After transfection, we harvested the 293-Cre cells similarly to other cultured cells used for Western blotting, and used these cell lysates from the transfected 293-Cre cells as appropriate immunoblotting technical controls.

For LXR/RXR agonist treatments involving measuring mRNA expression in treated iMAEC, cells were initially plated into six well tissue culture dishes containing standard growth medium, and grown to 70–80% confluency. We rinsed iMAEC with PBS and treated cells with either vehicle-only, the LXR agonist 22(R)-hydroxycholesterol and RXR agonist 9-cis-retinoic acid (both at 10 μM final concentration), or the LXR agonist GW3965 and RXR agonist SR11237 (both at 10 μM final concentration), all in basal conditions. After treating the cells for 24 h, we rinsed cells with PBS, and isolated and quantified the total RNA from these treated iMAEC to use for RT-qPCR. We also treated another group of iMAEC with LPS (Sigma-Aldrich) to use as a technical positive control, since LPS induces pro-inflammatory expression in cultured endothelial cells [24,61]. We maintained, handled, and processed this group of iMAEC similarly to the vehicle and LXR/RXR agonist treated cells used for our RT-qPCR studies, but substituted the vehicle/agonist treatment with LPS (100 ng/mL) [31].

### 4.3. Immunoblotting

Cell lysate preparation and Western blotting was performed as previously described [37]. Briefly, we first rinsed cells with PBS and harvested cells using RIPA lysis buffer containing mammalian protease inhibitors (VWR Life Science). We quantified the amount of protein within cell lysates via using a BCA assay kit (BioVision, Milpitas, CA, USA). Equal amounts of protein in the cell lysates were separated using SDS-PAGE and separated proteins were transferred onto PVDF membranes (Merck Millipore Ltd., Burlington, MA, USA). PVDF membranes were blocked in TBST buffer containing 5% (*w*/*v*) nonfat dry milk. Blots were probed with the following primary antibodies: mouse anti-ABCA1 (1:1000 dilution, sc-58219; Santa Cruz Biotechnology, Dallas, TX, USA); goat anti-LXR (1:2500 dilution, NB100-1465; Novus Biologicals, Littleton, CO, USA); mouse anti-RXR (1:1000 dilution, sc-46659; Santa Cruz Biotechnology); mouse anti-HSP90 (1:5000 dilution, 610419; BD Biosciences, San Jose, CA, USA); and mouse anti-GAPDH (1:1000 dilution, sc-365062; Santa Cruz Biotechnology). After probing with primary antibodies, we washed the blots with TBST, and incubated the blots with the following secondary antibodies: HRP-conjugated chicken anti-goat IgG secondary antibody (1:10,000 dilution, HAF019; Novus Biologicals) to recognize the goat anti-LXR primary antibody; and HRP-conjugated goat anti-mouse IgG secondary antibody (1:10,000 dilution, AP181P; Sigma-Aldrich) to recognize the mouse anti-ABCA1, anti-RXR, anti-GAPDH, and anti-HSP90 primary antibodies. We detected bound secondary antibodies by using ECL substrate reagent (Immobilon ECL Ultra Western HRP Substrate; MilliporeSigma, Billerica, MA, USA) and image analyses were conducted by use of a ChemiDoc system (Analytik Jena US, Upland, CA, USA). We quantified all signals within our blots by using NIH ImageJ software and the housekeeping proteins (i.e., loading controls) used for our immunoblots were GAPDH and HSP90.

### 4.4. Cholesterol Efflux Assays

To determine ABCA1 functionality in iMAEC, both iMAEC and pMAEC (positive control) were cultured in basal conditions in 48-well plates and grown to 70–80% confluency. We rinsed cells with PBS and loaded cells with [^3^H]cholesterol (1 μCi/mL;PerkinElmer, Waltham, MA, USA) diluted in high-glucose DMEM supplemented with 1% penicillin/streptomycin and 2 mg/mL of fatty acid-free bovine serum albumin (Sigma-Aldrich). Twenty-four hours later, we rinsed cells with PBS and refed cells with high-glucose DMEM supplemented with 1% penicillin/streptomycin and 2 mg/mL of fatty acid-free bovine serum albumin, either with or without apoAI (5 μg/mL; Academy Bio-Medical Company, Houston, TX, USA), for another twenty-four hours, before collecting cells and medium to measure apoAI-mediated cholesterol efflux.

For LXR/RXR agonist treatments, we cultured iMAEC in 48-well plates in basal conditions until cells reached 70–80% confluency. We rinsed iMAEC with PBS and refed cells with standard growth medium that contained either vehicle only, the LXR agonist 22(R)-hydroxycholesterol and RXR agonist 9-cis-retinoic acid (both at 10 μM final concentration), or the LXR agonist GW3965 and RXR agonist SR11237 (both at 10 μM final concentration), for 24 h. After LXR/RXR agonist and vehicle treatments, we rinsed iMAEC with PBS, and loaded cells with [^3^H]cholesterol (1 μCi/mL) diluted in high-glucose DMEM supplemented with 1% penicillin/streptomycin and 2 mg/mL of fatty acid-free bovine serum albumin. Twenty-four hours later, we rinsed iMAEC with PBS and refed cells with high-glucose DMEM supplemented with 1% penicillin/streptomycin and 2 mg/mL of fatty acid-free bovine serum albumin, either with or without apoAI (5 μg/mL; Academy Bio-Medical Company, Houston, TX, USA), for 24 hours, and collected medium and cells to be used for measuring apoAI-mediated cholesterol efflux. The 293-Cre technical control groups were treated in the same conditions as the vehicle and LXR/RXR agonist treated iMAEC by receiving the same volume of medium and the same concentration of [^3^H]cholesterol and apoAI. Medium from all cells used in our cholesterol efflux experiments was collected and then filtered via centrifuging the medium in filtration plates (2500× *g* for 10 min) to remove any detached cells floating in the medium. We rinsed [^3^H]cholesterol-loaded cells with PBS and lysed these cells by adding 250 μL of NaOH (200 mM) to each well and freeze-thawed the plates. The [^3^H] in the cellular extracts and medium samples were measured by using a liquid scintillation counter (LS 6500; Beckman Coulter, Brea, CA, USA). ApoAI-mediated cholesterol efflux was calculated by dividing [^3^H] in the medium by total [^3^H] (medium + cells), after subtracting background cholesterol efflux in cells not incubated with the cholesterol acceptor apoAI [31,35,37].

### 4.5. RT-qPCR

We measured mRNA cellular expression as previously described [37]. Briefly, cells were lysed with TRI reagent and total RNA was column-purified by a Direct-zol RNA purification kit (Zymo Research, Irvine, CA, USA). We quantified the isolated total RNA by using a SpectraMax QuickDrop spectrophotometer (Molecular Devices, LLC., San Jose, CA, USA). After quantifying the total RNA from all treated cells, we treated the total RNA with DNase I (Promega Corporation, Madison, WI, USA), and then converted 100 ng of total RNA into cDNA (qScript cDNA SuperMix; Quantabio, Beverly, MA, USA) to use as template for qPCR (PerfeCTa SYBR Green FastMix; Quantabio, Beverly, MA, USA). We normalized mRNA expression of the pro-inflammatory genes ICAM-1, VCAM-1, IL-6, and CCL2 by using GAPDH as the housekeeping gene. All sequences for the primer pairs used are listed in Table 1. We performed our qPCR reactions using a qTOWER³ G touch qPCR instrument (Analytik Jena US) and the qPCR data was analyzed by using the ΔΔ^CT^ method [62].

### 4.6. Statistical Analyses

SigmaPlot (Systat Software, Inc., San Jose, CA, USA) was used to perform statistical analysis. The normality test performed was a Shapiro–Wilk test. The equal variance test performed was a Brown–Forsythe test. When both normality and equal variance assumptions were met, a one-way ANOVA was performed. When normality was violated and/or equal variances were not assumed, a Kruskal–Wallis one-way ANOVA on ranks was performed. The post hoc analysis used for the Western blotting experiments and cholesterol efflux assays was a Dunnett’s test, while the post hoc analysis used for the mRNA expression experiments was a Dunn’s test. All results are described as mean ± SEM and significance was set at *p*-value of <0.05.

## Figures and Tables

**Table 1:** Primer pairs for RT-qPCR.

Target		Sequence (5′–3′)
GAPDH	forward:	TGACCTCAACTACATGGTCTACA
	reverse:	CTTCCCATTCTCGGCCTTG
VCAM-1	forward:	CCCGTCATTGAGGATATTGG
	reverse:	AATCTCAGGAGCTGGTAG
ICAM-1	forward:	CAATTTCTCATGCCGCACAG
	reverse:	AGCTGGAAGATCGAAAGTCCG
CCL2	forward:	GTCCCTGTCATGCTTCTG
	reverse:	CTGCTGGTGATCCTCTTG
IL-6	forward:	TCTATACCACTTCACAAGTCGGA
	reverse:	GAATTGCCATTGCACAACTCTTT

## Data Availability

The data presented in this study are available on request from the corresponding author. The data are not publicly available due to privacy.
